# Human EAG channels are directly modulated by PIP_2_ as revealed by electrophysiological and optical interference investigations

**DOI:** 10.1038/srep23417

**Published:** 2016-03-23

**Authors:** Bo Han, Kunyan He, Chunlin Cai, Yin Tang, Linli Yang, Stefan H. Heinemann, Toshinori Hoshi, Shangwei Hou

**Affiliations:** 1 Key Laboratory of Systems Biomedicine (Ministry of Education), Institute of Systems Biomedicine, Shanghai Jiao Tong University, Shanghai 200240, China; 2 Center for Molecular Biomedicine, Department of Biophysics, Friedrich Schiller University Jena & Jena University Hospital, Hans-Knöll-Str. 2, D-07745 Jena, Germany; 3 Department of Physiology, University of Pennsylvania, Philadelphia, PA 19104, USA; 4 Tongren Hospital, School of Medicine, Shanghai Jiao Tong University, Shanghai 200240, China; 5 State Key Laboratory of Oncogenes and Related Genes, Shanghai Cancer Institute, Renji Hospital, School of Medicine, Shanghai Jiao Tong University, Shanghai 200240, China

## Abstract

Voltage-gated e*ther à go-go* (EAG) K^+^ channels are expressed in various types of cancer cells and also in the central nervous system. Aberrant overactivation of human EAG1 (hEAG1) channels is associated with cancer and neuronal disorders such as Zimmermann-Laband and Temple-Baraitser syndromes. Although hEAG1 channels are recognized as potential therapeutic targets, regulation of their functional properties is only poorly understood. Here, we show that the membrane lipid phosphatidylinositol 4,5-bisphosphate (PIP_2_) is a potent inhibitory gating modifier of hEAG1 channels. PIP_2_ inhibits the channel activity by directly binding to a short N-terminal segment of the channel important for Ca^2+^/calmodulin (CaM) binding as evidenced by bio-layer interferometry measurements. Conversely, depletion of endogenous PIP_2_ either by serotonin-induced phospholipase C (PLC) activation or by a rapamycin-induced translocation system enhances the channel activity at physiological membrane potentials, suggesting that PIP_2_ exerts a tonic inhibitory influence. Our study, combining electrophysiological and direct binding assays, demonstrates that hEAG1 channels are subject to potent inhibitory modulation by multiple phospholipids and suggests that manipulations of the PIP_2_ signaling pathway may represent a strategy to treat hEAG1 channel-associated diseases.

The human *ether à go-go* channel (hEAG1, also known as Kv10.1, encoded by the gene KCNH1) is a voltage-gated K^+^ channel mainly expressed in neuronal and cancer cells^1^. Besides the well-established roles in tumor development^2^,^3^,^4^,^5^, the importance of hEAG1 channels in the nervous system is now increasingly appreciated. For instance, recent genetic studies have demonstrated that EAG1 channels are critical for shaping the action potential in mice^6^, and gain-of-function mutations of the channel are associated with Zimmermann-Laband and Temple-Baraitser syndromes, two severe neurological and developmental disorders^7^,^8^. The hEAG1 channel has promising therapeutic and diagnostic potential, and development of the channel inhibitors is one therapeutic strategy for treating cancer and neurological disorders^7^,^8^,^9^. However, regulation of the hEAG1 channel function is only beginning to be understood. Although a section of the C-terminal area of hEAG1 shares high sequence similarity with the cyclic nucleotide binding domain in cyclic nucleotide-gated (CNG) channels and hyperpolarization-activated cyclic nucleotide-modulated (HCN) channels^10^, several lines of evidence have demonstrated that the hEAG1 channel fails to bind cyclic nucleotides^11^,^12^,^13^. Only a few endogenous regulators of the hEAG1 channel have been identified^12^,^14^. Among them, Ca^2+^/calmodulin (CaM) potently inhibits the EAG1 channel by binding to up to three discrete intracellular areas located in its N and C termini^15^,^16^,^17^.

Phosphatidylinositol 4,5-bisphosphate (PIP_2_), a phospholipid composed of one negatively charged head group and two fatty acid tails, serves as a structural cofactor for many membrane proteins and it is the precursor of two important second messengers, diacylglycerol (DAG) and inositol 1,4,5-trisphosphate (IP_3_). Acting as a multifunctional molecule, PIP_2_ plays pivotal roles in normal and pathological cellular functions^18^,^19^,^20^. For instance, PIP_2_ is implicated in cell proliferation and neurological diseases^21^,^22^,^23^. Consequently, the lipid kinases including phosphatidylinositol 4-kinases (PI4Ks) and phosphatidylinositol-4-phosphate 5-kinases (PIP5KIs) responsible for PIP_2_ synthesis are considered to be potential therapeutic targets for various disorders involving altered neuronal excitability such as chronic pain^19^,^22^,^24^,^25^,^26^. In addition, the degradation of PIP_2_ is subject to dynamic regulation by many important neuronal transmitters via G protein-coupled receptor (GPCR)-induced phospholipase C (PLC) activation, which in turn affects many important downstream targets including a wide array of ion channels^19^,^27^,^28^. The mechanisms of ion channel regulation by PIP_2_ appear diverse. A crystal structure of an inward-rectifier K^+^ channel with PIP_2_ bound shows a direct interaction between the negatively charged head group of the lipid and a cluster of positively charged residues of the channel^29^. Besides the direct interaction mechanism, PIP_2_ also exerts its modulatory influences on ion channels indirectly by binding to numerous channel-associated inositol-binding proteins^30^,^31^,^32^. Moreover, modulation of ion channel function by PIP_2_ may involve alteration of the physical and chemical properties of the plasma membrane, which could then change the channel structure and function^33^. For instance, membrane lipid rafts enriched in PIP_2_^34^ may influence ion channels contained therein^35^,^36^. Despite the existence of these direct and indirect mechanisms, many studies investigating modulation of ion channels by PIP_2_ relied solely on electrophysiological measurements and mutagenesis, and failed to clarify the exact mechanism of the PIP_2_ action.

The potential importance of both hEAG1 channels and PIP_2_ in neurological and developmental disorders^7^,^8^,^9^ prompted us to investigate whether hEAG1 channels are regulated by PIP_2_. In this study, we show that gating of the hEAG1 channel is potently inhibited by exogenous PIP_2_ and that physiological depletion of endogenous PIP_2_ in the plasma membrane enhances the channel activity. Moreover, the bio-layer interferometry (BLI) assay demonstrates that PIP_2_ directly binds to the hEAG1 channel complex. Removal of a short domain located in the N terminus of the channel abolished its interaction with PIP_2_ as well as the lipid-induced ionic current inhibition. Our findings show that the hEAG1 channel is directly regulated by PIP_2_ and that this regulation may contribute to normal human physiology and pathology.

## Results

### Inhibition of hEAG1 channels by PIP_2_

**Figure 1:**
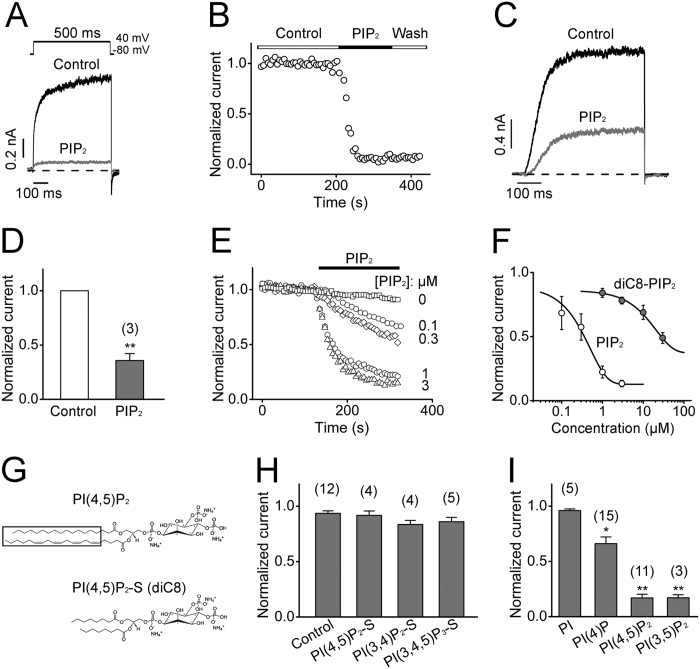
Inhibitory effects of PIP_2_ on hEAG1 channels. (**A**) Representative current traces recorded before and after application of brain-derived PIP_2_ (3 μM). Pulses to 40 mV were applied from the holding potential of −80 mV. (**B**) Representative time course of inhibition of normalized hEAG1 current by 3 μM PIP_2_ at 40 mV. Representative whole-cell hEAG1 currents (**C**) and normalized hEAG1 channel peak current (**D**) elicited by pulses to 40 mV from −80 mV using an automated patch-clamp instrument (Patchliner, Nanion) before and after intracellular perfusion of PIP_2_ (3 μM). **P < 0.01 compared to control levels before lipid applications. (**E**) Time course of normalized peak hEAG1 current at 40 mV after application of different concentrations of PIP_2_ (n = 5–13). (**F**) Concentration-dependent effects of PIP_2_ (open circles, n = 5–9) and diC8-PIP_2_ (closed circles, n = 3–7) (200 s applications) on the peak hEAG1 current size at 40 mV. The smooth curves were obtained by the Hill equation fitting. (**G**) Structures of long-chain and short-chain (S) phospholipids with the hydrophobic tails (long chain) framed. (**H**) Summarized normalized peak current of hEAG1 channels recorded at 3 min after application of diC8-PIP_2_ and its analogs (3 μM). The current size was normalized to that before lipid application in each patch. (**I**) Fractional peak hEAG1 current at 40 mV in the presence of the lipids indicated (3 μM). The numbers of independent measurements are shown in parentheses.

The effect of PIP_2_ on heterologously expressed hEAG1 channels was studied by direct application of exogenous PIP_2_ to the intracellular side of excised patches from Chinese hamster ovary (CHO) cells transiently expressing hEAG1 channels. Acute application of 3 μM brain-derived PIP_2_ with 18 to 20 carbon tail groups^31^ to the intracellular side caused a rapid and potent current inhibition (Fig. 1A,B). Because currents through hEAG1 channels frequently underwent progressive rundown after patch excision, our analysis was typically limited to the results obtained within 5–8 min of patch excision. We also tested the effect of PIP_2_ on the whole-cell current by perfusing PIP_2_ into the HEK293T cells stably expressing hEAG1 channels using a fast intracellular perfusion system and observed a similar inhibitory effect (Fig. 1C,D). The development of the inhibitory effect was slower with lower concentrations of PIP_2_ (Fig. 1E) and the results obtained with 200-s PIP_2_ application epochs showed that the fractional decrease in current size was also concentration dependent with an apparent half-maximal inhibitory concentration (IC_50_) of 0.35 ± 0.01 μM and the Hill coefficient of 0.92 ± 0.32 (Fig. 1F). By contrast, dioctanoyl-PIP_2_ (diC8- PIP_2_) and its analogs with different numbers of negative charges (3 μM), which have 8-carbon tails and higher water solubility^27^ than brain-derived PIP_2_, showed less overall potency in inhibiting hEAG1 channels (Fig. 1F–H). The IC_50_ value for diC8-PIP_2_ was 18.6 ± 0.6 μM, suggesting that the inhibitory potency of PIP_2_ depends on the length of the fatty acid chains.

In addition to PIP_2_, other types of phosphatidylinositol (PIs) are also involved in cellular signaling and they may show specificities in ion channel regulation^37^,^38^. We therefore tested whether these PI lipids inhibited the hEAG1 channel. When applied at 3 μM, PI, PI(4)P, PI(4,5)P_2_, and PI(3,5)P_2_ differed noticeably in their abilities to alter the hEAG1 current, from no effect by PI to the strongest inhibition by PIP_2_ and PI(3,5)P_2_ (Fig. 1I), showing that the number of negative charges of the head group is also critical for the channel inhibition.

**Figure 2:**
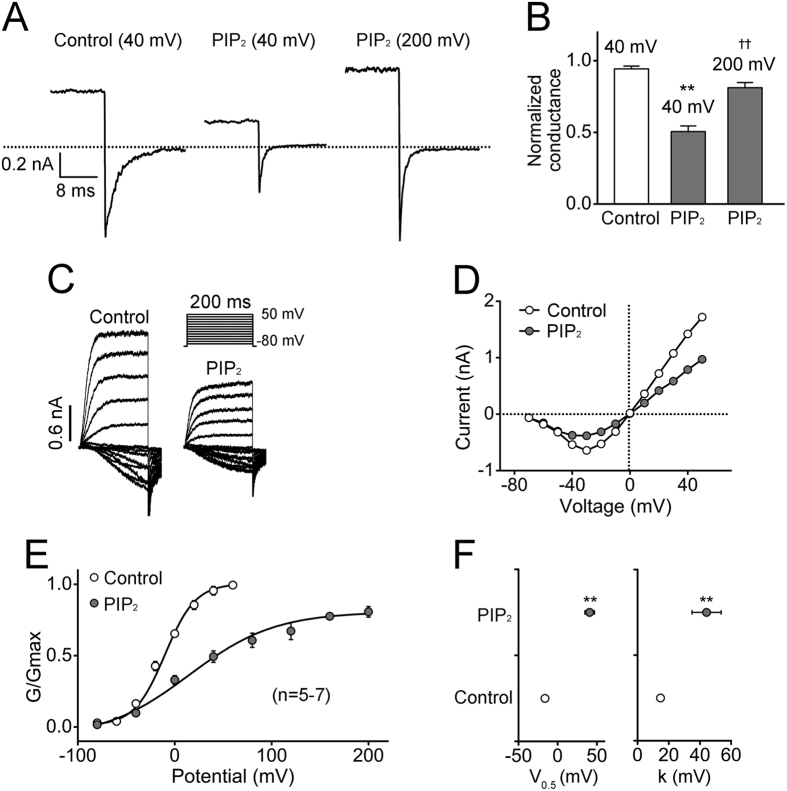
Voltage dependence of hEAG1 channel inhibition by PIP_2_. (**A**) Representative current traces were recorded at −80 mV following depolarization to different voltages indicated. PIP_2_ was applied at 2 μM. (**B**) Normalized conductance under the conditions shown in (**A**). The results obtained in the presence of PIP_2_ (2 μM) were normalized to the maximal conductance inferred from the tail currents before PIP_2_ application in each patch. The normalized conductance values were 0.94 ± 0.02, 0.51 ± 0.04 and 0.81 ± 0.04, respectively (n  =  6). (**C**) Illustrative current traces elicited by pulses from −80 to 50 mV in 10 mV increments before and after application of 0.1 μM PIP_2_. (**D**) Peak current-voltage curves from the results shown in (**C)**. (**E**) Normalized conductance-voltage (GV) curves constructed from hEAG1 channel tail currents before and 3–5 min after application of 0.1 μM PIP_2_. The curves were fitted with a Boltzmann function from the results obtained using the same protocol as in (**C**). The fit parameters, half-activation voltage (V_0.5_) and slope factor (k), of voltage-dependent activation of hEAG1 channels before and after PIP_2_ application (0.1 μM) are shown in (**F**). **P < 0.01 compared to the control before PIP_2_ application and ^††^*P* < 0.01 compared to after PIP_2_ application at 40 mV.

PIP_2_ (3 μM) virtually annihilated currents through hEAG1 at 40 mV at which the channel was maximally activated (Fig. 1A). However, even with PIP_2_ present (2 μM), depolarization to an extremely positive voltage (e.g., 200 mV) did elicit a robust outward current and the peak tail current size following the depolarization was similar to that after depolarization to 40 mV without PIP_2_ (Fig. 2A,B). This observation suggests that hEAG1 remained functional even in the presence of 3 μM PIP_2_ (Fig. 1A) but the voltage dependence of activation was shifted markedly to the positive direction. Consistent with this possibility, the conductance-voltage (GV) curve obtained with a lower concentration of PIP_2_ (0.1 μM) showed a clear positive shift; the half-activation voltage (V_0.5_) changed from −16.1 ± 4.0 to 40.8 ± 6.1 mV (Fig. 2E,F). Additionally, the GV curve became less steep; the slope factor (k) increased from 14.7 ± 1.2 to 44.2 ± 9.3 mV (Fig. 2C–F).

### hEAG1 channels interact directly with PIP_2_

**Figure 3:**
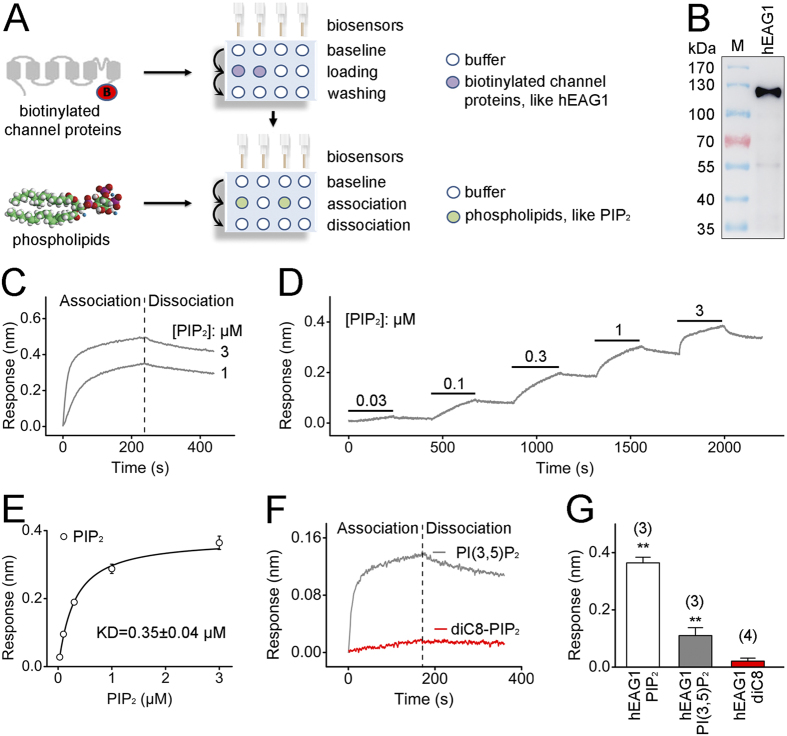
hEAG1 channels directly interact with PIP_2_. (**A**) A schematic diagram showing the BLI binding assay protocol. (**B**) Western blot detection of hEAG1 channel protein from purified protein samples. The anti-FLAG antibody used recognizes a single protein band of ~110 kDa consistent with full-length of FLAG-tagged hEAG1 channel. (**C**) Changes in optical interference showing the dynamic association and dissociation processes between the channel protein and PIP_2_ at the concentrations indicated. The dashed line denotes the time at which the BLI sensors were transferred to the control buffer. The raw traces of association and dissociation were fitted well using double-exponential functions. For the association component, the time constants for the fast and slow component were 7.90 ± 0.04 s and 81.7 ± 1.2 s, respectively. For the dissociation segment, the time constants for the fast and slow component were 4.53 ± 0.67 s and 128.8 ± 3.5 s, respectively. (**D**) Changes in optical interference in different concentrations (0.03–3 μM) of PIP_2_ in a representative assay. (**E**) Curve fit with Hill equation obtained from the peak value of the optical interference signal measured at different PIP_2_ concentrations for determination of the equilibrium dissociation constants (KD) of the interaction between the hEAG1 channel protein and PIP_2_ (n = 3). (**F**) Representative time courses of the interference signals elicited by PI(3,5)P_2_ and diC8-PIP_2_. Each was applied at 3 μM. Data are representative of three separate experiments. (**G**) Optical interferences signal changes by 3 μM of different species of phospholipids. Numbers of independent measurements are shown in parentheses. **P < 0.01 compared to control before PIP_2_ application.

To test whether PIP_2_ directly interacts with the hEAG1 channel, we used bio-layer interferometry (BLI) to measure the kinetics of binding of PIP_2_ to the purified hEAG1 channel complex. BLI is a novel methodology that has emerged recently for detecting protein-protein interactions as well as those between proteins and small molecules. Binding events between a protein and its ligands can be measured by changes in optical interference (Fig. 3A). The quality and the specificity of purified biotinylated hEAG1 channel protein was confirmed by Western blot (Fig. 3B). Addition of PIP_2_ to the protein anchored to the BLI sensor tip rapidly increased the optical interference signal (Fig. 3C). The kinetics of the BLI signal resembled those observed in the patch-clamp measurements in the excised patches (Fig. 1B). The kinetics of the increase in the BLI signal by PIP_2_ was concentration dependent, becoming faster with greater concentrations, and the BLI signal increase was partially reversible after 3 min of wash (Fig. 3C,D). Using a 200-s application epoch at each concentration as utilized in the electrophysiological experiments, the cumulative concentration dependence suggested a dissociation constant (KD) value of 0.35 ± 0.04 μM (Fig. 3E), similar to the IC_50_ value obtained from the electrophysiological measurements (Fig. 1F).

The close correspondence between the result from the BLI and electrophysiological measurements was also observed in the ligand selectivity. PI(3,5)P_2_ (3 μM), which effectively inhibited the hEAG1 channel electrophysiologically (Fig. 1I), elicited robust BLI signals (Fig. 3F,G). In contrast, diC8-PIP_2_ (3 μM), which failed to inhibit the hEAG1 channel in our electrophysiological experiments (Fig. 1G,H), also failed to elicit any noticeable BLI signal (Fig. 3F,G).

### Depletion of endogenous PIP_2_ increases hEAG1-mediated K^+^ currents

**Figure 4:**
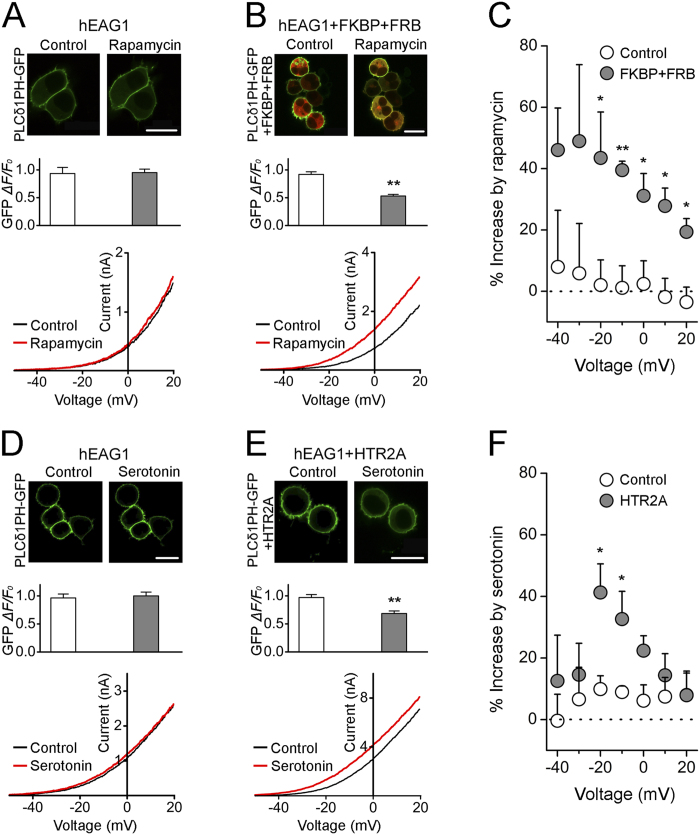
Enhancement of hEAG1 channel current by PIP_2_ hydrolysis induced by rapamycin and HTR_2A_ activation. (**A**) Representative GFP fluorescence (top), normalized GFP fluorescence at the plasma membrane (middle), and whole-cell current traces (bottom) in hEAG1-expressing cells transfected with PLCδ1PH-GFP in the absence and presence of rapamycin (100 nM) for 30 s as indicated. (**B**) Representative fluorescence (top), the normalized GFP fluorescence at the plasma membrane (middle), and the whole-cell current (bottom) in hEAG1-expressing cells transfected with PLCδ1PH-GFP, 5-phosphatase-FKBP-mRFP (FKBP), and PM-FRB-CFP (FRB) before and after incubation with rapamycin (100 nM) for 30 s. (**C**) Rapamycin (100 nM)-induced fractional augmentation of hEAG1 current at different voltages from hEAG1-expressing cells transfected with PLCδ1PH-GFP alone (n = 5) and with PLCδ1PH-GFP, 5-phosphatase-FKBP-mRFP, and PM-FRB-CFP (n = 3). (**D**) Representative GFP fluorescence (top), the normalized GFP fluorescence at the plasma membrane (middle), and the whole-cell current in hEAG1-expressing cells transfected with PLCδ1PH-GFP before and after incubation with serotonin (100 μM) for 1 min. (**E**) Representative fluorescence (top), the normalized GFP fluorescence at the plasma membrane (middle), and whole-cell current traces (bottom) in hEAG1-expressing cells transfected with PLCδ1PH-GFP (PH) and the serotonin receptor HTR_2A_ (HTR2A) in the absence and presence of serotonin (100 μM) for 1 min. (**F**) Serotonin-induced fractional increase of hEAG1 current at different voltages from hEAG1-expressing cells transfected with PLCδ1PH-GFP alone (n = 5) and PLCδ1PH-GFP and HTR_2A_ together (n = 6). *P < 0.05 and **P < 0.01 compared to the control. Scale bars in (**A**,**B**,**D**,**E**) 20 μm. ∆F/F_0_ represents the normalized GFP fluorescence intensity in the plasma membrane after PIP_2_ depletion. The currents were elicited by 1 s-ramps from −80 to 20 mV every 5 s.

Some inward-rectifier K^+^ channels require PIP_2_ for function, indicating that these channels are subject to basal tonic modulation by PIP_2_^20^. We investigated whether a similar tonic modulation of hEAG1 channels exists by selectively depleting PIP_2_ in HEK293T cells stably expressing hEAG1 channels. We used the plasma membrane-targeted, rapamycin-induced translocation system based on inducible dimerization of PM-FRB-CFP and 5-phosphatase-FKBP-mRFP to acutely deplete PIP_2_ by a specific dephosphorylation of PIP_2_ at the 5′ position^39^,^40^. PIP_2_ depletion in the plasma membrane was monitored by PLCδ1PH-GFP, which binds to PIP_2_ at the plasma membrane^40^. When hEAG1-expressing cells were transfected with PLCδ1PH-GFP alone, the GFP fluorescence was found primarily near the plasma membrane (Fig. 4A top and middle), suggesting an appreciable level of PIP_2_ in the plasma membrane at rest. Application of 100 nM rapamycin to these cells changed neither the location of GFP fluorescence distribution nor the electrophysiologically measured whole-cell hEAG1 current size (Fig. 4A bottom, C). By contrast, the same concentration of rapamycin diminished the GFP signal when 5-phosphatase-FKBP-mRFP and PM-FRB-CFP constructs were cotransfected with PLCδ1PH-GFP (Fig. 4B top and middle), indicative of a decrease in the level of plasma membrane PIP_2_. Rapamycin also increased the whole-cell hEAG1 current (Fig. 4B bottom). The fractional increase in current was especially noticeable at more negative voltages (Fig. 4C), consistent with the idea that PIP_2_ depletion shifted the current-voltage (IV) relationship to the negative direction.

One of the physiological ways by which PIP_2_ is hydrolyzed is by the action of phospholipase C (PLC) following activation of GPCRs such as serotonin receptors^41^, which are in turn implicated in tumorigenesis^42^ and also in neuronal signaling. We thus examined if serotonin increases the hEAG1 channel activity by depleting PIP_2_. We transfected the hEAG1-expressing HEK293T cells with human 5-hydroxytryptamine (serotonin) receptor 2A (HTR_2A_)^43^ and PLCδ1PH-GFP. Hydrolysis of PIP_2_ by PLC leads to IP_3_ release and increases the intracellular Ca^2+^ concentration ([Ca^2+^]_i_), which may confound any effect of PIP_2_ depletion^16^. To avoid this potential complication, the “fast” Ca^2+^ chelator 1,2-bis(o-aminophenoxy)ethane-*N*,*N*,*N′*,*N′*-tetraacetic acid (BAPTA, 10 mM) was used in the whole-cell recording solution to rapidly control [Ca^2+^]_i_. Serotonin (100 μM) had no detectable effect on the GFP signal or the current size in the control hEAG1-expressing cells (Fig. 4D,F). However, when the channels were co-expressed with the serotonin receptor HTR_2A_, serotonin dimmed the GFP signal reporting the PIP_2_ level (Fig. 4E top and middle) and noticeably increased the whole-cell current (Fig. 4E bottom, F). The fractional increase in current was greater at more negative voltages (Fig. 4F), suggesting that the IV curve shifted to the negative direction. The results collectively suggest that endogenous PIP_2_ exerts a detectable tonic inhibitory influence on hEAG1 channels in intact cells.

### Molecular determinants of hEAG1 channel inhibition by PIP_2_

**Figure 5:**
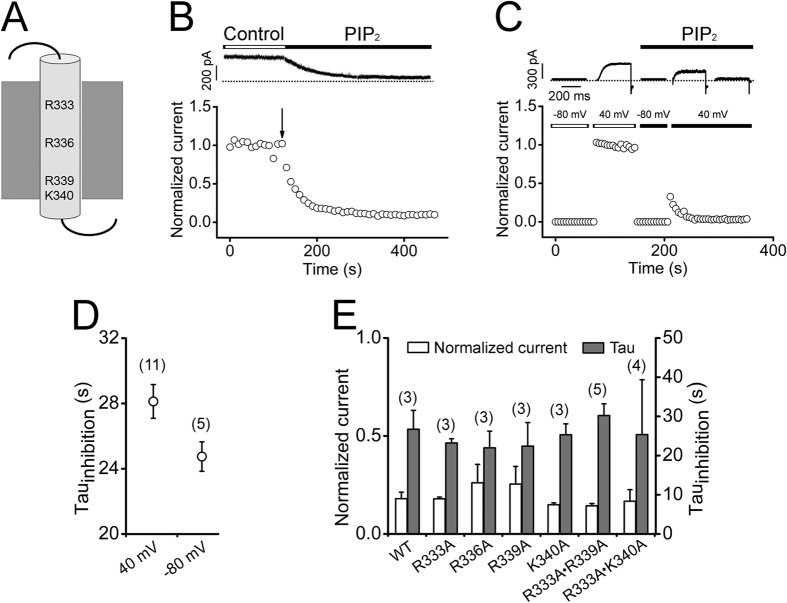
Positive charges in the S4 voltage sensor are not essential for the inhibitory effect of PIP_2_. (**A**) A cartoon showing the positively charged amino acids in hEAG1 S4. (**B**) Representative current trace (top) and the normalized time course (bottom) of hEAG1 channel currents in an excised patch before and after application PIP_2_ (3 μM) when the membrane potential was held at 40 mV. The arrow denotes the start of PIP_2_ application. (**C**) Representative current traces recorded sequentially at −80 mV, 40 mV, −80 mV, and 40 mV (top), and the normalized time course (bottom) at the membrane voltages indicated. (**D**) Averaged time constants of PIP_2_-induced hEAG1 current inhibition at 40 and −80 mV according to the protocols in (**B**,**C**) respectively. (**E**) Summary of the normalized peak current and averaged time constants (Tau) of PIP_2_-induced current inhibition at 40 mV in wild-type and mutant hEAG1 channels. The time constants were obtained from single-exponential fits to the normalized current inhibition by PIP_2_.

PIP_2_-binding sites typically consist of positively charged amino acid residues^44^. To identify the molecular locus required for the PIP_2_-dependent regulation of hEAG1, we first investigated whether the positively charged residues in S4 of hEAG1 are involved in PIP_2_-induced channel inhibition in part because PIP_2_ alters the GV characteristics of the hEAG1 channel (Fig. 2E). The hEAG1 S4 segment, like those in other voltage-gated K^+^ channels^45^,^46^, contains multiple positively charged residues that move within the electric field (Fig. 5A). If these mobile charges interact with PIP_2_ applied to the intracellular side, some voltage/S4 state dependence of the effect of PIP_2_ on hEAG1 may be expected as observed in KCNQ channels^47^. PIP_2_ quickly inhibited the hEAG1 channel when the membrane potential was held at 40 mV (Fig. 5B,D). At this voltage, assuming that hEAG1 and Kv1.2/2.1^46^ activate in a similar fashion, the voltage sensors are expected to be fully activated, placing hEAG1 K327 through R336 on the extracellular side and R339 interacting with negatively charged residues in S2 and S3. A similar inhibition was observed when the membrane potential was held at −80 mV at which R330 through K340 electrically should face the intracellular side (Fig. 5C,D). Furthermore, neutralization of the positively charged residues at R333, R336, R339, and/or K340 had no appreciable effect on the PIP_2_-induced channel inhibition (Fig. 5E).

**Figure 6:**
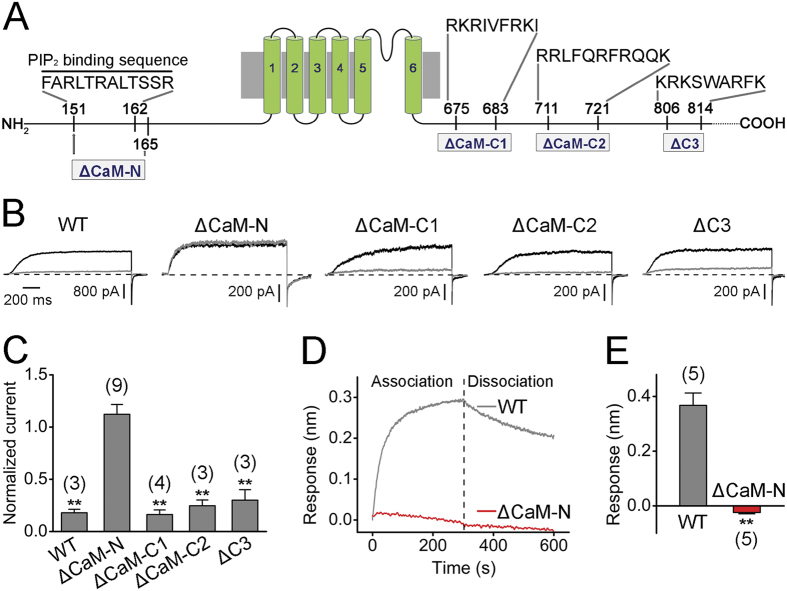
Deletion of the N-terminal CaM binding site impairs the PIP_2_-mediated inhibition of hEAG1. (**A**) Structural organization of one hEAG1 subunit. The putative PIP_2_ binding domain, CaM-N, CaM-C1, CaM-C2, and C3 are illustrated. (**B**) Representative current traces recorded at 40 mV before (black) and after application (gray) of 3 μM PIP_2_ in wild-type and the mutant channels. (**C**) Summary of the normalized peak current at 40 mV after application of PIP_2_ (3 μM) in wild-type and mutant hEAG1 channels. **P < 0.01 compared to before PIP_2_ application. (**D**) Kinetics of PIP_2_ (3 μM) interaction with wild-type (gray) and ∆CaM-N (red) hEAG1 channels using the BLI assay. (**E**) Mean optical interference responses obtained 300 s after application of PIP_2_ (3 μM) for wild-type and ∆CaM-N hEAG1 channel proteins. **P < 0.01 compared to WT. Numbers of independent measurements are shown in parentheses.

Amino-acid sequences of hEAG1, hELK1, and hERG1 show considerable similarity (~40%). A recent study has shown that PIP_2_ inhibits hELK1 by interacting with some of the positively charged residues in the S4–S5 linker, S6, and EAG domain^48^. However, these residues are not well conserved in hEAG1 channels. By contrast, PIP_2_ increases currents through hERG1 and a short region rich in positively charged residues (^883^RQRKRKLSFRRR^894^) in the intracellular C terminus of hERG1 has been implicated^49^. The distal C terminus of hEAG1 also contains a similar segment with multiple positively charged residues (C3 ^806^KRKSWARFK^814^, Fig. 6A). Deletion of this segment did not alter the inhibitory effect of exogenous PIP_2_ (∆C3, Fig. 6B). In many ion channels such as KCNQ^50^, TRPC^51^, SK2^52^, and CNG channels^53^, the putative PIP_2_ interaction sites overlap with CaM binding sites. Therefore, we hypothesized that the CaM interaction domains in the hEAG1 channel^15^,^16^,^17^ may be involved in the inhibitory effect of PIP_2_. Up to three potential CaM binding segments in hEAG1 have been reported: CaM-N in the N terminus and CaM-C1 and CaM-C2 in the C terminus (Fig. 6A)^15^,^16^,^17^. We deleted these CaM binding sites individually (∆CaM-N, ∆CaM-C1 and ∆CaM-C2). Currents through hEAG1 ∆CaM-C1 and hEAG1 ∆CaM-C2 maintained wild-type-like sensitivity to PIP_2_; however, those from hEAG1 ∆CaM-N were virtually unaltered by PIP_2_, up to 3 μM (Fig. 6B,C). Moreover, no BLI signals were observed from the purified hEAG1 ∆CaM-N in response to PIP_2_ (3 μM; Fig. 6D,E). In addition to hEAG1, we also found that PIP_2_ significantly inhibited hEAG2 (KCNH5, Kv10.2) channels, which have virtually the same sequence near the CaM-N area as hEAG1 (Supplementary Fig. S1).

### Functional independence between PIP_2_ and Ca^2+^/CaM in the regulation of hEAG1 channels

**Figure 7:**
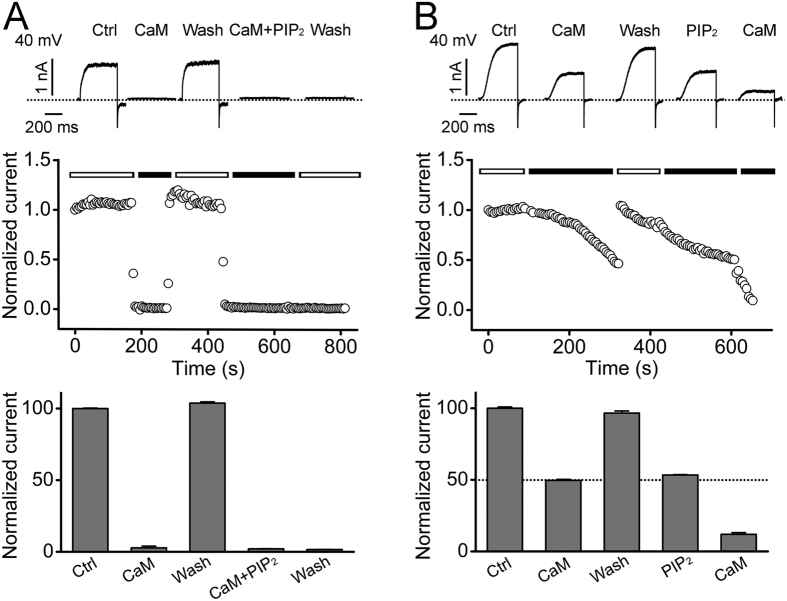
CaM- and PIP_2_-induced hEAG1 channel inhibition. (**A**) Representative current traces (top), the time course of the normalized peak current (middle) and the summarized normalized peak current (bottom) of hEAG1 channels recorded at 40 mV in excised patches before and after sequential applications of CaM (200 nM) and PIP_2_ (3 μM) as indicated (n = 4). (**B**) Representative current traces (top), the time course of the normalized peak current (middle) and the summarized current inhibition (bottom) of hEAG1 channels recorded at 40 mV in excised patches before and after sequential applications of CaM (30 nM) and PIP_2_ (0.5 μM), both of which induced around 50% current inhibition as shown by the dotted line (n = 3). The internal Ca^2+^ was buffered to 1 μM in both (**A**,**B**).

The observation that the potential binding sites for PIP_2_ and Ca^2+^/CaM in the N terminus overlap suggests that these two inhibitory factors may interfere with each other. Furthermore, both Ca^2+^/CaM and PIP_2_ may be present together under physiological conditions. The exposure of inside-out membrane patches to 200 nM CaM in 1 μM [Ca^2+^]_i_ resulted in a complete inhibition of hEAG1 currents and this effect, unlike that by PIP_2_, was rapidly and fully reversible (Fig. 7A). The second exposure to Ca^2+^/CaM at the same concentrations together with PIP_2_ (3 μM) inhibited the current as rapidly as the first exposure. However, no recovery was observed after wash out of Ca^2+^/CaM (Fig. 7A), an indication of the long-lasting action of PIP_2_ (see Fig. 1). To assess whether the presence of PIP_2_ alters its sensitivity to Ca^2+^/CaM, we utilized the following protocol. We first verified that Ca^2+^/CaM (30 nM CaM in 1 μM [Ca^2+^]_i_) inhibited the current in a rapidly reversible fashion (Fig. 7B, first “CaM”). Following wash out of Ca^2+^/CaM, application of PIP_2_ (0.5 μM) decreased the current by 50% (Fig. 7B, “PIP_2_”). Subsequent application of Ca^2+^/CaM results in a near 100% inhibition (Fig. 7B, second “CaM”), suggesting that Ca^2+^/CaM and PIP_2_ may have additive effects on hEAG1 channel inhibition.

## Discussion

Increasing evidence suggests that hEAG1 K^+^ channels are closely linked with tumorigenesis and developmental diseases^7^,^8^,^9^. However, the regulation of the hEAG1 channel function is only beginning to be elucidated and only a few physiologically relevant regulators have been identified^12^,^14^,^16^,^54^ . Here we have demonstrated that PIP_2_ profoundly shifts the voltage dependence of activation of the hEAG1 channel to the positive direction and acts as a potent inhibitory modulator of the channel. Further, we have identified that a region near the channel’s N-terminal CaM interaction site is essential for both the electrophysiological effects and binding of PIP_2_.

Membrane lipids may be an important class of modulators of EAG channels. A recent study has shown that mouse neurons have two populations of EAG channels, one of which is sensitive to the alteration of lipid raft integrity such as cholesterol depletion^54^. In our study, temporally-controlled depletion of PIP_2_ by rapamycin-induced translocation of 5-phosphatase^55^ in whole cells noticeably increased hEAG1 currents especially at negative voltages. Thus, PIP_2_-mediated inhibition of hEAG1 channels is well suited to influence action potential firing in neurons. Physiological relevance of the tonic inhibitory influence of PIP_2_ as an endogenous modulator of hEAG1 is further suggested by the finding that activation of serotonin HTR_2A_ receptors, known to activate PLC^43^ to promote hydrolysis of PIP_2_, also increases hEAG1 currents at negative voltages. However, the magnitude of hEAG1 current increase *in vivo* mediated by GPCR activation is difficult to predict. Under such conditions, GPCR activation leads to generation of multiple signaling molecules capable of affecting hEAG1 channels in distinct ways. For example, PIP_2_ depletion by PLC produces IP_3_, which in turn can promote Ca^2+^ release from the endoplasmic reticulum. Ca^2+^/CaM could then inhibit hEAG1 channels, dampening the direct stimulatory effect of PIP_2_ depletion on the channels described here. In addition, activation of many GPCRs including serotonin receptors can stimulate phospholipases A2 (PLA2) thereby inducing the release of arachidonic acid (AA), a lipid messenger which has been reported to activate hEAG channels in human melanoma cells^14^. Thus, depending on the comparative potencies and kinetics of these direct and indirect effects, complex regulation of hEAG1 channels by PIP_2_ under physiological conditions is possible.

Numerous ion channels have been reported to be PIP_2_ sensitive^20^. However, elucidation of the exact mode of action of PIP_2_, for example direct binding vs. indirect effects, has been hampered by the lack of appropriate PIP_2_ binding assays with a good time resolution that detect physiologically relevant binding events. Here, we used bio-layer interferometry (BLI), a novel assay capable for measuring the kinetics of binding between proteins and their ligands^56^, to detect binding of PIP_2_ to the isolated hEAG1 protein. The BLI measurements showed the existence of two kinetic components in association and dissociation (Fig. 3C). The fast BLI association and slow dissociation components especially well correspond to the electrophysiological results in their kinetics and concentration dependence. The slow BLI association component and the fast dissociation signals, which became more prominent at high concentrations of PIP_2_, may be electrophysiologically silent. Furthermore, the deletion of the N-terminal Ca^2+^/CaM binding area in hEAG1, which eliminates the electrophysiological effect of PIP_2_, also abolishes the BLI signal. A Hill coefficient (~1) of the concentration dependence curve of the current inhibition by PIP_2_ hints that binding of one PIP_2_ is capable of exerting a full inhibitory effect; this idea needs further investigation. The close correspondence between the electrophysiological and BLI results demonstrates that BLI, which requires no labeling and has a time resolution comparable to that of the patch-clamp method, could be a very promising method for studying the pharmacology of ion channels, thus complementing the established electrophysiological assays.

**Figure 8:**
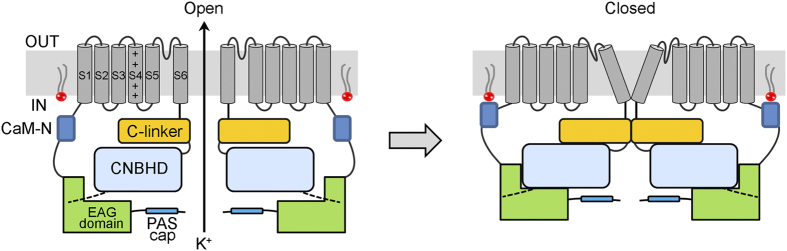
Proposed mechanism of PIP_2_ inhibition in hEAG1 channels. A cartoon of two subunits of a tetrameric hEAG1 channel complex shown in the open (left) and closed (right) states. PIP_2_ (red) binding to the CaM-N segment (blue) may cause a conformational change of the complex formed by EAG domain and CNBHD and then close the channel gate via the C-linker region as described in Haitin *et al.*^13^.

Our electrophysiological results show that PIP_2_ functions as a potent inhibitory gating modifier of the hEAG1 channel by shifting the overall voltage dependence of activation to the positive direction. We did not measure the exact shift at high concentrations of PIP_2_; however, the observation that a functionally saturating concentration of PIP_2_ (3 μM) essentially eliminates the current at 40 mV where the normalized conductance without PIP_2_ is near unity suggests that PIP_2_ may induce a >100 mV shift. Such a large shift in the voltage dependence of activation may partially explain the profound current inhibition. How binding of PIP_2_ to the N terminus of hEAG1 leads to this marked shift in the overall voltage dependence can only be speculated. Studies show that the N-terminal region of the members of the KCNH channel family (K_V_10, K_V_11 and K_V_12) including hEAG1 (K_V_10.1) plays pivotal roles in channel gating^57^. An area termed the EAG domain in the N terminus directly interacts with an area in the C terminus called the cyclic nucleotide-binding homology domain (CNBHD), which in turn regulates behavior of the ion conduction gate in S6 via the C-linker domain^13^. Downstream of the EAG domain close to S1, the N terminus contains one of the CaM binding sites, CaM-N, which is involved in Ca^2+^/CaM-induced inhibition of hEAG1^17^. Our results show that the amino-acid residues involved in PIP_2_ binding partially overlap with the CaM-N site, suggesting that PIP_2_ and Ca^2+^/CaM may interact. Such an interaction is in fact seen in many other ion channels such as TRPC6 in which PIP_2_ binding disrupts the association of CaM with the channel protein^51^. A structural study shows that PIP_2_ activates type 2 small-conductance Ca^2+^-dependent K^+^ channels (SK2) by binding to the positively charged residues located at the interface of the SK2-CaM complex and that phosphorylation of CaM reduces the affinity of PIP_2_ for the channel complex^52^. However, in hEAG1, the long-lasting inhibitory effect of PIP_2_ is unaltered by Ca^2+^/CaM while we cannot formally exclude the possibility that binding of PIP_2_ somehow renders the interaction of the CaM-N domain and CaM essentially irreversible. Thus, PIP_2_ and Ca^2+^/CaM with vastly different kinetics are capable of regulating the channel in each other’s presence, expanding the modulatory versatility of the hEAG1 channel. The N terminus of the EAG channel has been suggested to interact directly with CNBHD^13^. Perhaps, this interaction is modified by PIP_2_ to alter the voltage dependence of activation (Fig. 8).

In summary, we have demonstrated that PIP_2_ is a potent endogenous gating modulator of the hEAG1 channel that exerts its tonic inhibitory influence by direct binding to the N terminus of the channel protein. Gain-of-function mutations in the gene coding for hEAG1 have been associated with developmental and neurological disorders such as Zimmermann-Laband and Temple-Baraitser syndromes^7^,^8^. Thus, the tonic inhibition of the hEAG1 channel by PIP_2_ may be beneficial to the aforementioned disorders and dysregulation of the PIP_2_ signaling pathway may contribute to disease through hEAG1 channels.

## Materials and Methods

### Channel expression

hEAG1 (NM_002238.3) and hEAG2 (NM_139318.4) in the expression vector pcDNA3.1 were transiently expressed in CHO cells using X-tremeGENE 9 (Roche Diagnostics). The mutant channels were constructed by using the QuickChange Site-Directed Mutagenesis kit (Agilent) following the manufacturer’s instruction.

### Purification and biotinylation of FLAG-tagged channel proteins

Wild-type and mutant channels with a FLAG tag at the distal C terminus were prepared in the pCDH lentiviral expression plasmid (System Bioscience). To generate cells stably expressing hEAG1 or the mutant channels, ~2.45 μg of the DNA construct was transfected into 5 × 10^5^ HEK293T cells using the calcium phosphate transfection method in a 35 mm diameter tissue-culture dish. The cells were cultured in DMEM (Invitrogen) supplemented with 10% FBS (Invitrogen) and 5 μg/ml puromycin (Invitrogen). After 2 days of culture, cells were trypsinized and seeded onto 10 cm diameter tissue-culture plates. The cells were passaged every 2–3 days and supplied with fresh puromycin 5 μg/ml contained selection medium for 12–14 days. Puromycin-resistant cells were trypsinized and seeded onto 96-well tissue-culture dishes at a density of ~1 cell per 2 wells. Monoclonal puromycin-resistant cells were cultured and expanded to prepare the seed stocks for future use and concomitantly to electrophysiologically examine the channel expression. To purify hEAG1 wild-type and the mutant channel proteins, cells stably expressing the channel of interest were harvested from DMEM containing 2 μg/ml puromycin, and were solubilized in a lysis buffer (10 mM HEPES, 1.5 mM MgCl_2_, 10 mM NaCl, 1% NP-40, pH 7.0) for 30 min followed by sedimentation by centrifugation (15,000 × g, 30 min). The supernatant was collected and mixed with anti-FLAG beads (Sigma) overnight. The beads-captured protein was collected based on the manufacturer’s instruction. All steps were performed at 4 °C or on ice and all buffers contained Complete Protease Inhibitor (Roche Diagnostics). The purified proteins were quantified using BCA kit (Biyuntian, China) and labeled with biotin (Pierce). The biotinylated membrane proteins were stored in the buffer (PBS with 0.02% Tween-20 and 0.1% BSA, pH 7.4) for the bio-layer interferometry (BLI) assay.

### Western blot

The purified hEAG1 protein samples (2 μg) were separated by 8% SDS-polyacrylamide gel and then transferred to nitrocellulose membranes. After blocking with 5% nonfat milk in Tris-buffered saline containing 0.1% Tween-20 (TBST) for 1 h at room temperature, the transferred membranes were incubated overnight at 4 °C with M2 monoclonal anti-FLAG antibody (1: 1000; Sigma), followed by a goat anti-mouse HRP-conjugated secondary antibody (1: 5000; Santa Cruz Biotechnology). The ECL chemiluminescence was visualized and captured by GE Amersham Imager 600 Imaging System.

### Bio-layer interferometry (BLI)

Binding of phospholipids to the channel protein was measured and analyzed on an Octet Red96 instrument (FortéBio) at room temperature. The buffer-equilibrated streptavidin biosensors were loaded with 100 μg/ml protein. A duplicate set of sensors was incubated in the buffer without protein for a background binding control. The assay was performed in black 96-well plates (Thermo Fisher Scientific) with the total working volume of 0.21 ml per well. The signal was analyzed using a double reference subtraction protocol to subtract the non-specific binding, background, and signal drift caused by sensor variability. The binding event between the protein and the lipids was quantified by the shift of interference pattern of the light^58^.

### Electrophysiology and analysis

Macroscopic currents were recorded in the inside-out patch-clamp configuration from CHO cells transiently expressing hEAG1 channels by an EPC-10 amplifier (HEKA Electronics) controlled by PatchMaster software (HEKA Electronics). Patch pipettes pulled from borosilicate glass (Warner) had a typical initial resistance of 1 to 3 MΩ when filled with the pipette solution described below and ~60% of the initial input resistance was electronically compensated in the macroscopic current measurements. Leak and capacitative currents were subtracted with a P/6 protocol. The external solution contained (in mM): 140 KCl, 2 MgCl_2_, 10 HEPES, 15 glucose, pH 7.2 with *N*-methyl-*D*-glucamine (NMG). The internal solution without Ca^2+^ contained (in mM): 140 KCl, 10 EGTA, 10 NaCl, 1 MgCl_2_ and 10 HEPES, pH 7.2 with NMG. Internal solutions with higher concentrations of free Ca^2+^ were prepared using various amounts of EGTA as described previously^59^. The current was elicited to different voltages from the holding potential of −80 mV every 2 s. The lipids were applied to the intracellular side when the current reached a stable level after patch excision. Half-activation potential (V_0.5_) was obtained by fitting the maximal peak tail current versus membrane voltage with a Boltzmann function,


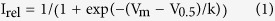


where V_m_ is the membrane potential, and k is the slope factor.

Whole-cell currents were recorded from HEK293T cells stably expressing wild-type or mutant hEAG1 channels using an EPC10 amplifier and in some experiments using an automated planar patch-clamp instrument (Nanion) as described previously^60^. The external solution contained (in mM): 134 NaCl, 6 KCl, 2 CaCl_2_, 1 MgCl_2_, 10 glucose, 10 HEPES, pH 7.4 (with NMG). The intracellular solution contained (in mM): 110 K aspartate, 30 KCl, 10 NaCl, 2 MgCl_2_, 10 HEPES, pH 7.2 (with NMG).

Automated whole-cell electrophysiological measurements were conducted according to Nanion’s standard procedure with 8-channel Patchliner (Nanion) equipped with an EPC-10 quadro patch-clamp amplifier (HEKA Electronics). Single-use borosilicate glass chips with medium resistance (1.8 to 3 MΩ, NPC-16, Nanion) were used for all recordings. The PatchControlHT (Nanion) and PatchMaster (HEKA Electronics) were used for cell capture, seal formation, whole-cell access, and subsequent recording in the voltage-clamp configuration. The internal solution contained (in mM): 50 KCl, 60 KF, 10 NaCl, 20 EGTA, and 10 HEPES, pH 7.2 (KOH). The external solution contained (in mM): 140 NaCl, 4 KCl, 2 CaCl_2_, 5 glucose, and 10 HEPES, pH 7.4 (NaOH). Currents were elicited by voltage steps from the holding potential of −80 mV to 40 mV every 2 s. All electrophysiological experiments were performed at room temperature. Data analysis and curve fitting were done using FitMaster (HEKA Electroncis) and figures were prepared with Origin 8.5 (OriginLab).

### Confocal imaging

HEK293T cells were cultured on glass coverslips and transfected with the various constructs, PH(PLCδ1)-GFP, 5-phosphatase-FKBP-mRFP and PM-FRB-CFP^55^ (generous gifts from T. Balla) using X-tremeGENE 9 (Roche Diagnostics) and cultured for 24 h. Live-cell dual-color measurements were performed on a spinning-disc confocal microscope (Nikon). Excitation lasers were processed with appropriate filter sets for GFP and RFP to capture cellular fluorescence images. Images were captured at room temperature and processed by the NIS elements imaging software (Nikon).

### Reagents

Brain-derived PIP_2_ was from Sigma. All other phospholipids were obtained from Avanti. Serotonin, rapamycin, and calmodulin were purchased from Sigma. Stock solutions of phospholipids (1 mM) were prepared in deionized H_2_O and stored in glass vials at −20 °C and diluted to the final concentrations immediately before experiments by vigorous vortexing as described previously^38^. Unless otherwise noted, brain-derived PIP_2_ was used in the study.

### Statistical analysis

Data are expressed as mean ± SEM. Statistical significance was evaluated using either an unpaired *t*-test or one-way ANOVA followed by Student-Newman-Keuls’s multiple comparisons test as appropriate. Statistical significance of *P* < 0.05 and *P* < 0.01 is indicated by single and double asterisks or daggers, respectively.

## Additional Information

**How to cite this article**: Han, B. *et al.* Human EAG channels are directly modulated by PIP_2_ as revealed by electrophysiological and optical interference investigations. *Sci. Rep.* **6**, 23417; doi: 10.1038/srep23417 (2016).

## Supplementary Material

Supplementary Information
