# Supplementary material for: Human EAG channels are directly modulated by PIP2 as revealed by electrophysiological and optical interference investigations
Source: Sci Rep. 2016 Mar 23;6:23417. doi: 10.1038/srep23417 (PMC4804213; doi:10.1038/srep23417)
Supplement: Supplementary Information [file srep23417-s1.pdf]

Supplementary Information

Human EAG channels are directly modulated by PIP_2 as revealed by electrophysiological and optical interference investigations

Bo Han[1], Kunyan He[1], Chunlin Cai[1], Yin Tang[1], Linli Yang[1], Stefan H. Heinemann[2], Toshinori Hoshi[3], Shangwei Hou[1,4,5,*]

[1]Key Laboratory of Systems Biomedicine (Ministry of Education), Institute of Systems Biomedicine, Shanghai Jiao Tong University, Shanghai 200240, China

[2]Center for Molecular Biomedicine, Department of Biophysics, Friedrich Schiller University Jena & Jena University Hospital, Hans-Knöll-Str. 2, D-07745 Jena, Germany

[3]Department of Physiology, University of Pennsylvania, Philadelphia, PA 19104, USA

[4]Tongren Hospital, School of Medicine, Shanghai Jiao Tong University, Shanghai 200240, China

[5]State Key Laboratory of Oncogenes and Related Genes, Shanghai Cancer Institute, Renji Hospital, School of Medicine, Shanghai Jiao Tong University, Shanghai 200240, China

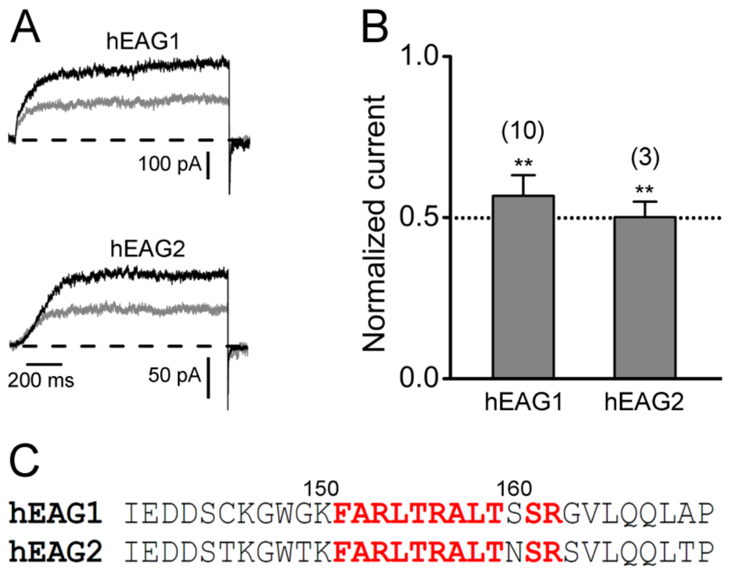

Figure S1: Both hEAG2 and hEAG1 channels are inhibited by PIP2. (**A**) Representative current traces of hEAG1 and hEAG2 channels before and 30 s

following application of 3 μM PIP2. The currents were elicited by pulses to 40 mV from a holding potential of –80 mV. (**B**) Normalized hEAG1 channel peak current before and after PIP2 (3 μM) application. $P < 0.01$ compared to the control level before PIP2 applications (Statistical significances were evaluated by one-way ANOVA with Student-Newman-Keuls's multiple comparisons test). (**C**) Sequence alignment of the N termini of hEAG1 and hEAG2 channels using ClustalW (http://www.ebi.ac.uk/Tools/msa/clustalw2/). The residues shown in red indicate the conserved residues that are key determinants of PIP2 inhibition on hEAG1 channels as shown in Figure 6.
